# The Coupling Synergy Effect of Economic and Environment in Developed Area: An Empirical Study from the Yangtze River Delta Urban Agglomeration in China

**DOI:** 10.3390/ijerph19127444

**Published:** 2022-06-17

**Authors:** Yongqiang Yin, Zhenxiao Xu

**Affiliations:** 1 School of Economics, Hangzhou Normal University, Hangzhou 311121, China; yinyongqiang7@163.com; 2 Alibaba Business School, Hangzhou Normal University, Hangzhou 311121, China

**Keywords:** Yangtze River Delta urban agglomeration, economic development, ecological environment, gray correlation, coupling, coordination

## Abstract

Coordination between economic development (ED) and ecological environment (EE) is an important challenge for high-quality urban development. Taking the panel data related to the ED and EE of the Yangtze River Delta urban agglomeration (YUA) from 2009 to 2019 as the research objective, the evaluation system of ED and EE was constructed by introducing the coupling coordination degree model and the gray correlation degree model to analyze their development indices, coupling coordination degree and gray correlation degree in two spatial and temporal dimensions. Research results: (1) The ED indexes and EE indexes of the 26 cities in the YUA have obvious differences in different cities, and there is no synergy between the two indices. (2) The coupling coordination degree of the YUA shows a pattern of high in the east and low in the west, high in the center and low in the north and south in space, and an overall increasing trend in time. (3) In terms of gray correlation, the correlation between ED quality dimension and EE level dimension is the highest. According to the conclusion, when both the economy and environment present consistency at a high level, it will help the city’s economy to develop more efficiently and rapidly.

## 1. Introduction

As the world economy develops into a new stage, people are increasingly stressing about the establishment of a modern economic system with harmony between human and nature, as well as high-quality and sustainable economic development. China has made astonishing achievements in urbanization in the past decades. However, many problems have also emerged, making the cities become “sub-healthy”. The contradiction between economy and environment in some cities is becoming increasingly prominent, resource guarantee is meeting its bottleneck and eco-environment protection is faced with problems. In this regard, the Chinese government has gradually taken measures to effectively promote urban sustainable development. In 2012, the 18th National Congress of the Communist Party of China put forward the strategic goal of the five-sphere integrated plan and raised the construction of ecological civilization to a higher strategic level. In 2017, the 19th National Congress of the Communist Party of China pointed out that we should adhere to the harmonious coexistence of man and nature and establish and practice the idea that green water and green mountains are the golden hills. In 2020, Xi Jinping put forward the integration and high-quality strategic objectives of the development of the Yangtze River Delta at the symposium on solid advancement of integration.

The YUA embraces the open urban belt along the eastern coast of China and the industrial intensive belt along the Yangtze River. It is the most dynamic and competitive economic agglomeration area in China. During the rapid urbanization and industrialization, however, more resources have been consumed, and the discharge of industrial waste is gradually increasing, plus the severe regional environmental pollution, bringing constant challenges to eco-environment. In this condition, the paper has assessed the coordination between ED and EE of YUA, which is of paramount importance for balancing regional ED and EE, so as to realize the high-quality development of the YUA.

Promoting the coordinated development of the economy and environment and improving sustainable efficiency are important connotations of high-quality urban development. Effectively measuring the coordination of the economy and environment and the correlation of internal factors are the prerequisites for evaluating the quality of urban development. The study area of this paper includes 26 cities in the Yangtze River Delta urban agglomeration, namely Nanjing, Wuxi, Changzhou, Suzhou, Nantong, Yancheng, Yangzhou, Zhenjiang and Taizhou in Jiangsu Province, Hangzhou, Ningbo, Jiaxing, Huzhou, Shaoxing, Jinhua, Zhoushan and Taizhou in Zhejiang Province, Hefei, Wuhu, Maanshan, Tongling, Anqing, Chuzhou, Chizhou and Xuancheng in Anhui Province, and Shanghai, the municipality directly under the Central Government. Then, we collect different indicators of the economy and environment to construct the evaluation system of ED and EE. The grey correlation model is used to measure the correlation degree between different indicators and subsystems, and the coupling coordination model is used to measure the coordination degree between economic development and ecological environment of urban agglomeration as a whole and at the provincial and municipal levels. Based on the empirical results, we put forward corresponding measures for the coordinated development of the economy and environment in the YUA and to improve the quality of urbanization development from the perspective of urban agglomeration as a whole and single city.

This paper is divided into five parts. The first part is the introduction; the second part is literature review; the third part introduces the research data and research methods; the fourth part measures the correlation between each index and the system; the fifth part analyzes the coordinated development level of provinces, cities and urban agglomerations from the aspects of development index, coupling degree and coordination degree, and further discusses the factors affecting the economy and environment. The sixth part summarizes the conclusions of this paper and puts forward some policy recommendations.

## 2. Literature Review

Urbanization has become a comprehensive problem of social development in contemporary China, which is a unique process [1]. The imbalance in the development has caused many social and environmental problems [2]. Two issues have been paid attention to by people in this process: the development of the city itself and the influence of urbanization on the eco-environment [3]. Urbanization includes four aspects, of which the most fundamental and important consequence is the improvement of urban economic level. There is an interactive coupling relationship between ED and EE [4]. On the one hand, it has a certain degree of stress effect on EE and a mitigation effect on environmental pressure. On the other hand, EE can effectively undertake and offset the negative effects brought by ED to a certain extent. However, the deterioration of ecology will also lower the quality of ecology and even restrict EE by repelling population inflows and external investment.

International studies stress the sustainable development of economy and environment. In 1987, the World Commission on Environment and Development put forward the concept of sustainable development for the first time in its report “Our Common Future” and described a series of economic and environmental problems human being were faced with [5]. Since then, as an important part of sustainable development, the coordinated development of the two has rapidly become a hot field of study on economy, environment and geography. Ecological modernization theory [6] believes that although economic development will damage the environment, the degree of this damage will be reduced in the process of development. On the contrary, monotonous production theory believes that the strong relationship between ED and EE will remain unchanged or even weaken with time. Through empirical studies, Jorgenson [7] believes that both the theories have some defects in explaining the structural relationship between economic development and environmental hazards, which need to be further improved to comprehensively explain the dynamic economic development and its environmental impact. Kemp-Benedict [8] used the new Ricardian model to prove the inverted pyramid relationship between the economy and environment, that is, natural resources that may only account for a small proportion of GDP have constituted the bottom of the inverted pyramid, while other parts of the economy have been distributed at the top of the pyramid. Other scholars from Europe [9], the United States [10], Singapore [11], Tanzania [12], Pakistan [13] and other countries and regions have carried out studies and found that the improvement of environmental quality in the future would be highly related to economic structure, and an optimized relationship between the economy and environment would be of great help to sustainable development.

Chinese scholars have discussed the correlation between ecological civilization and a circular economy and their construction path. Song Tao [14] explained the short-term dependent relationship and long-term equilibrium relationship between economic growth and pollution by metrological inspection. Zhu Dajian [15] believed that China should separate the economic and social development and the resource and environment consumption. Other scholars have taken Beijing–Tianjin–Hebei urban agglomeration [16], Yellow River Basin [17], Shenyang Economic Zone [18] and Yellow Sea Economic Belt [19] as research objects and claimed that the construction of ecological civilization shall be integrated into economic development.

The research methods of regional economic development and eco-environment mainly include the Grey Models [20], Data Envelopment Analysis (DEA) [21,22], Environmental Kuznets Curve (EKC) [23,24], Coupled Coordination Degree Model [25,26,27], Remote Sensing and ArcGIS Technology [28], etc. Compared with other methods, the Coupled Coordination Degree Model can characterize the interaction among multiple systems and reflect the coordinated relationship and situation among them [29]. Scholars use coupling coordination models to study different scales and disciplines, such as economy [30], ecology [31], tourism [32], land [33], urbanization [34], urban resilience [35], etc.

To sum up, current researches on regional development level mainly focus on provincial and municipal administrative regions, and less attention has been paid to urban agglomerations. In the limited studies on urban agglomerations, most of them only carried out data calculation and elaboration, less analysis from the “agglomeration” aspect. Therefore, by using the grey relational model and coupling coordination degree model, the paper has determined the coupling coordination correlation between the ED and EE of YUA and its spatial and temporal evolution law.

## 3. Indicator System and Research Methods

### 3.1. Indicator System

By referring to existing research results [36,37], 10 indicators were selected from ED scale, potential and quality in the ED subsystem, 9 indicators were selected from EE level, pressure and protection in the EE subsystem. The total of 19 indicators are selected to construct the evaluation index system for the coordinated development of ED and EE in the YUA (Table 1). Data in the paper were collected from China statistics bureaus at all levels and Statistical Yearbooks.

### 3.2. Data Processing

Considering the difference of original data in type and magnitude, the data were processed to be compared with different years and regions. Firstly, the original data were processed by range standardization, and the case was avoided when the base was 0 in the calculation of entropy by logarithm. The standardized data were uniformly multiplied by 0.99 and then added by 0.01. The calculation formulas are as follows:

(1) $$U_{\mathrm{ij}}=\frac{X_{\mathrm{ij}}-\min\left(X_{\mathrm{ij}}\right)}{\max\left(X_{\mathrm{ij}}\right)-\min\left(X_{\mathrm{ij}}\right)}\times 0.99+0.01,\;U_{\mathrm{ij}}\;\mathrm{is}\;a\;\mathrm{positive}\;\mathrm{indicator}$$

(2) $$U_{\mathrm{ij}}=\frac{\max\left(X_{\mathrm{ij}}\right)-X_{\mathrm{ij}}}{\max\left(X_{\mathrm{ij}}\right)-\min\left(X_{\mathrm{ij}}\right)}\times 0.99+0.01,\;U_{\mathrm{ij}}\;\mathrm{is}\;a\;\mathrm{negative}\;\mathrm{indicator}$$

Calculate the proportion of the j-th indicator in the i-th year

(3) $$p_{\mathrm{ij}}=\frac{U_{\mathrm{ij}}}{\sum_{i=1}^{m}U_{\mathrm{ij}}}$$

Calculate the information entropy value of the j-th indicator

(4) $$e_{j}=-\frac{1}{\ln m}\sum_{i=1}^{m}p_{\mathrm{ij}}\ln p_{\mathrm{ij}}$$

Calculate the difference coefficient of the j-th indicator

(5) $$a_{j}=1-e_{j}$$

Calculate the weight of the j-th indicator

(6) $${\;w}_{j}=\frac{a_{j}}{\sum_{i=0}^{n}a_{j}}$$

Establish the comprehensive development index of ED and EE subsystem in the i-th year

(7) $$v_{i}=\sum_{j=1}^{n}w_{j}U_{\mathrm{ij}}$$

(8) $$U_{1}=\sum_{j=1}^{n}w_{j}U_{\mathrm{ij}}^{X},U_{2}=\sum_{j=1}^{n}w_{j}U_{\mathrm{ij}}^{Y}$$

### 3.3. Research Methods

#### 3.3.1. Grey Relational Model

Based on the grey system theory and by establishing the grey relational model, the paper has calculated the correlation coefficient ξ_(m, n) and relational degree R_(m, n) and obtained the grey relational degree between the subsystem and evaluation indicators in the two systems, so as to determine the relational degree and coupling effect of each dimension of the subsystem of the former system and each evaluation indicator with those of the other system.

(9) $$\xi_{m,n}=\frac{\begin{array}{c} \min\\ m \end{array}\begin{array}{c} \min\\ n \end{array}\left|X_{\mathrm{ij}}-Y_{\mathrm{ij}}\right|+\rho \begin{array}{c} \max\\ m \end{array}\begin{array}{c} \max\\ n \end{array}\left|X_{\mathrm{ij}}-Y_{\mathrm{ij}}\right|}{\left|X_{\mathrm{ij}}-Y_{\mathrm{ij}}\right|+\begin{array}{c} \max\\ m \end{array}\begin{array}{c} \max\\ n \end{array}\left|X_{\mathrm{ij}}-Y_{\mathrm{ij}}\right|}$$

(10) $$R_{m,n}=\frac{1}{N}\sum_{m,n=1}^{N}\xi_{m,n},\left(m,n=1,\;2,3,\cdots ,N\right)$$

where $\xi_{m,n}$ and $R_{m,n}$ represent the correlation coefficient and relational degree between the m-th indicator of ED subsystem and the n-th indicator of EE subsystem of YUA in a certain year, respectively; $\begin{array}{c} \max\\ m \end{array}\begin{array}{c} \max\\ n \end{array}\left|X_{\mathrm{ij}}-Y_{\mathrm{ij}}\right|$ and $\begin{array}{c} \min\\ m \end{array}\begin{array}{c} \min\\ n \end{array}\left|X_{\mathrm{ij}}-Y_{\mathrm{ij}}\right|$ represented the two-stage maximum absolute difference and minimum absolute difference between the two levels; resolution coefficient ρ = 0.5. $R_{m,n}$ ranged from [0,1]. The larger the value, the stronger the coupling effect. Table 2 has shown the value of relational degree and classification standard.

#### 3.3.2. Coupling Coordination Degree Model

The coupling degree indicates the degree of interaction between multiple systems, and the coordination degree indicates the size of the benign relationship in these interactions. Based on this, this paper constructs the coupling coordination degree model of the economic development and ecological environment system:

(11) $$C={\left[\frac{\prod_{i=1}^{n}U_{i}}{{\left(\frac{1}{n}\sum_{i=1}^{n}U_{i}\right)}^{n}}\right]}^{\frac{1}{n}}$$

When n = 2,

(12) $$C={\left[\frac{U_{1}*U_{2}}{{\left(\frac{U_{1}+U_{2}}{2}\right)}^{2}}\right]}^{\frac{1}{2}}=\frac{\sqrt[{2}]{U_{1}U_{2}}}{U_{1}+U_{2}}$$

where C represents the coupling degree between the ED subsystem and EE subsystem of the YUA, which ranges from [0,1]. The closer C is to 1, the higher the degree of coupling between the two subsystems, and vice versa. Coupling can only indicate the degree of correlation between systems but cannot reflect the overall effectiveness and synergies between systems. Therefore, it is necessary to further construct the coupling coordination degree model to judge the coordination degree and synergy effect between systems. The calculation formula is as follows:

(13) $$T={\alpha U}_{1}+{\beta U}_{2}$$

(14) $$D=\sqrt{C*T}$$

T represents the comprehensive evaluation function; D represents the coupling degree and coordination between two subsystems; U_1_ and U_2_ represent the comprehensive development index of ED subsystem and EE subsystem, respectively; α and β refer to undetermined coefficients, which represent the contribution degree of ED and EE to the whole system, respectively. On the basis of consulting experts’ opinions and drawing on relevant research results, considering that their contributions are equal to each other, α = β = 0.5 is taken.

The paper has referred to the classification standard of coupling coordination degree in existing studies [38,39] to classify the coordinated development of economy and environment into 3 major categories and 10 minor categories according to the degree of their coupling degree and coordinated development degree, and the undertaking intervals are correspondingly shown.

## 4. Calculation and Analysis of Grey Relational Degree

### 4.1. Comparison between the Grey Relational Degrees of the Two Systems

Based on the grey relational degree model, the relational degree between the indicators and subsystems of the ED system and the EE system of the YUA in 2019 have been calculated and listed in Table 3 and Table 4.

In Table 5, the relational degree of indicators in the ED system and EE system are all above 0.5 except the growth rate of the total retail sales of consumer goods, the centralized treatment rate of urban sewage and the rate of harmless disposal of urban household garbage, which are important factors influencing the two systems. In sequence one, the tertiary industry accounted for the proportion of GDP correlation that is the highest, 0.639. It shows that the industrial structure is the most important factor affecting the level of ecological environment in the YUA, which is also in line with the corresponding situation of industrial structure and environmental state in the YUA. For the proportion of tertiary industry to GDP in the 26 cities, central cities have higher proportion than the north and south cities, and eastern cities are higher than western cities, which corresponds to the distribution of the coupling coordination degree.

In sequence two, the relational degree of the discharge of industrial wastewater, industrial smoke and dust emissions and industrial sulfur dioxide emissions are the top 3 indicators, indicating that the degree of industrial pollutant emissions has a great influence on urban ED in the YUA. In some cities of the YUA, heavy industries with high pollution have contributed a lot to economic growth, while some cities may face short-term industrial vacancy and economic downturn due to industrial upgrading.

### 4.2. Comparison between the Grey Relational Degrees of the Subsystems

Based on the calculation of the relational degree of each indicator, the sub-systems are calculated, respectively, and the relational degree and the grades of ED and EE subsystems of the YUA in 2019 have been obtained, as shown in Table 4.

Horizontally, EE level > EE pressure > EE protection, and the average relational degree of EE level is the highest, which is 0.6718. Vertically, the quality of ED > the scale of ED > ED potential, but there is not much difference among them. Therefore, the future development direction of the YUA is still to ensure the quality of economic development, improve the level of ecological environment and achieve high-quality development.

In terms of the relational degree between the subsystems, it is concluded that: ① The relational degree between ED scale and EE level is high, which is 0.6782. This is because the YUA belongs to one of the largest economic clusters in China, which needs a corresponding EE for its ED. ② The relational degree between ED potential and EE pressure is high, which is 0.6652. This is the case for the direct relation between the discharge of industrial pollution and the industrial structure. When the urban industrial structure is adjusted and upgraded, extensive enterprises are gradually eliminated and transferred, and the ecological pressure decreases. The construction of emerging industries has improved the development potential of the city. The construction of new industries will promote the ED potential of the city. ③ The relational degree between the quality of ED and EE level is high, which is 0.7156. This is because the quality of ED is mainly reflected in the per capital indicator. When the urban economic level is improved and the construction of ecological space is enhanced, the corresponding per capital EE level will also be improved.

## 5. Analysis of the Calculation Results of Coupling Coordination Degree

### 5.1. Development Index

**Figure 1:**
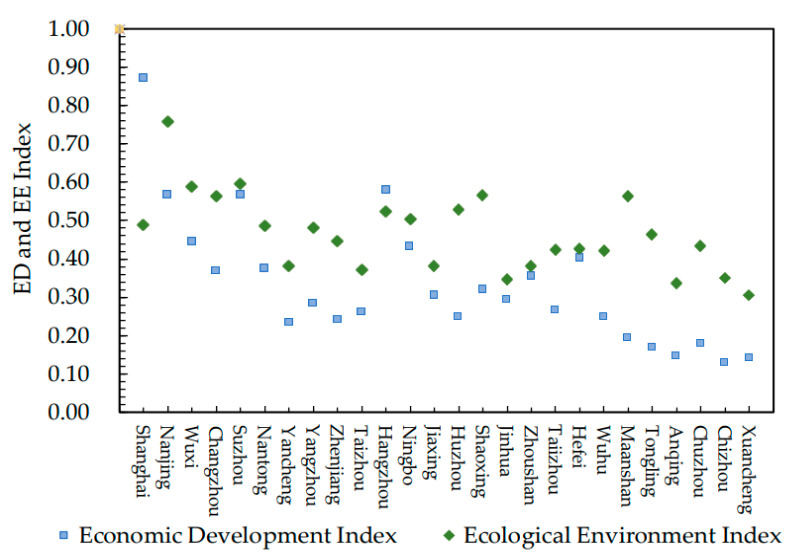
ED and EE Index of 26 Cities in YUA in 2019.

Based on Equation (8), the ED index and EE index of the 26 cities were determined, and the results of 2019 were selected to specifically analyze the development level and the types of economy and environment in each city, as shown in Figure 1.

**Figure 2:**
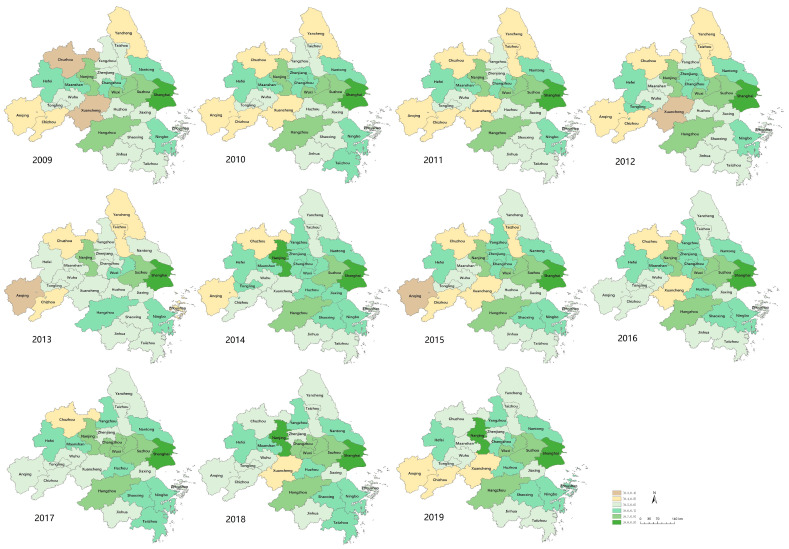
Spatial Distribution of ED and EE Coordination in YUA from 2009 to 2019.

Figure 2 has shown that the ED indexes are not consistent with the EE indexes in the 26 cities, and there is obvious difference. Based on the distribution of the two indicators in each city, the cities were classified into three categories as follows:

(1) EE lagging type. Shanghai is an example of this type. As the central city of the YUA, its ED level is much higher than that of most cities, becoming one of the objective reasons for its leading in ED index and lagging in EE. Although Shanghai has a relatively high EE index comparing with other cities, this index has not been coordinated with the ED. In future development, the construction of EE should be enhanced to coordinate it with the ED level.

(2) Balanced development type. The examples are Nanjing, Suzhou, Hangzhou, Ningbo, Jiaxing, Jinhua, Zhoushan and Hefei. These cities can be divided into two categories. One is with balanced development at a high level, such as Nanjing, Suzhou, Hangzhou, Ningbo and Hefei. The GDP of these cities has reached or surpassed a trillion. Meanwhile, as the representative cities in the agglomeration, city managers have been actively responding to the call of the state to promote the improvement of urban ecological level. For instance, Suzhou put forward the urbanization development path with the core concept of improving urban quality. The other category is coordinated development at a medium level, such as Jiaxing, Jinhua and Zhoushan, which all belong to Zhejiang Province. Restricted by geographical location and starting time, the ED level of these cities is relatively low. Affected by the radiation effect of central cities, however, these cities can positively embrace industrial transformation, learn from developed cities and thus have great development potential.

(3) ED lagging type. All the cities other than the above two types belong to this type, among which Wuxi, Changzhou, Nantong, Yancheng, Yangzhou, Zhenjiang, Taizhou, Huzhou, Shaoxing, Taizhou and Wuhu have a relatively high ED level, while Maanshan, Tongling, Anqing, Chuzhou, Chizhou and Xuancheng have relatively low ED. Most of the former cities belong to China’s major economic provinces—Jiangsu Province and Zhejiang Province. After the ED has reached a certain level, economy has in turn contributed to and improved the EE level in these cities. The latter cities belong to Anhui Province. As the interior region of the YUA and due to the siphonic effect of the developed cities along the eastern coast, resources, such as capital, labor and high-end enterprises, in these cities are relatively scarce, and the ED level is relatively low in these cities.

### 5.2. Coupling Degree

Based on Equation (12), the coupling degrees of the 26 cities of the YUA from 2009 to 2019 were determined, and the results have shown that the coupling degree C was concentrated on [0.8311,0.9999], all of which were in the high-level coupling interval. A total of 271 of the 286 C values were greater than 0.9, indicating that the interaction degree between the economy and environment in the 26 cities was extremely high and stable. From 2009 to 2019, the coupling degree was at a high level with an upward trend, suggesting that the coupling was virtuous and developing in a coordinated trend. In future urbanization, when the coupling degree of the two systems is gradually increasing, approaching or equaling one, a virtuous coupling between the economy and environment in the 26 cities will be achieved.

### 5.3. Coupling Coordination Degree

#### 5.3.1. Characteristics of Temporal and Spatial Evolution at the City Level

The coupling coordination degree of the ED and EE of the 26 cities in the YUA from 2009 to 2019 were calculated by coupling coordination degree model (Table 6), and the values were classified according to the classification system and discrimination standard of the coupling coordination degree.

In Table 4, D is distributed among [0.3602,0.8692], most of which belong to transitional development and coordinated development. Only a few years in Anqing, Chuzhou and Xuancheng belong to the imbalance and decline category, and the degree of decline is mild. Then, the paper has used ArcGIS10.6 for visualization based on the determination and has drawn the spatial distribution map of the coupling coordination degree between the ED and EE of the YUA from 2009 to 2019 (Figure 2).

In general, the coupling coordination degree between the ED and EE of the YUA had an upward trend from 2009 to 2019, while some of the cities had some backwards. In 2009, the cities that were barely coordinated accounted for 54% of the total, and other types of cities were scattered. In 2019, cities that were barely and primarily coordinated accounted for 69% of the total, and there were no cities with imbalance and decline. Spatially, cities in the eastern and central areas had higher coupling coordination degrees than those in western, northern and southern areas, with significant spatial heterogeneity.

Specifically, ① High-quality coordination has not yet appeared, showing that the coordination between the ED and EE of cities in the YUA has not yet reached an optimum degree, and the coordination mode of promoting environmental protection by economic development and promoting economic development with high-quality ecology has not yet been fully achieved. ② Coordinated development type: only Shanghai, Nanjing, Wuxi, Suzhou, Hangzhou and Ningbo had coordinated development in all the past 11 years, among which Shanghai is way ahead in the coordination score and has maintained a coordinated development. In the YUA, cities that belong to this type mainly are distributed in the central and eastern coastal areas because of their superior geographical location, early starting time and rapid development. In addition, when the economical level in these cities reaches a certain level, it in turn contributes to the eco-environment. Therefore, the ED and EE in these cities had always maintained a high level and their comprehensive development score is higher than that of other cities. ③ Cities that belong to the type from transitional development to coordinated development include Changzhou, Nantong, Yangzhou, Zhenjiang, Jiaxing, Huzhou, Shaoxing, Jinhua, Taizhou, Hefei, Wuhu, Ma’anshan and Tongling, accounting for 50% of the total. These cities are in different transitional stages. Only a few years of development in Changzhou, Nantong and Hefei belong to the transitional development type, and coordinated development will be achieved in these cities in the near future. However, Jiaxing, Taizhou, Wuhu, Tongling and other cities have only a few or no years of coordinated development, showing that although these cities have implemented corresponding strategies, they started late and no significant effects has been made yet. ④ Cities that belong to the type of transition from imbalance and decline to transitional development include Yancheng, Taizhou, Zhoushan, Anqing, Chuzhou, Chizhou and Xuancheng, among which Zhoushan belonged to the imbalance and decline only in 2013, and Chuzhou was only in the transitional stage in 2018 and 2019. The number of cities that belongs to this type is shrinking, and they are gradually gathering in Anhui Province, showing that some cities in Anhui Province have undertaken industrial transfer from Jiangsu, Zhejiang and Shanghai to strengthen their own economic development in the previous period, but they have also suffered from high pollution and high energy consumption brought by these transferred industries. ⑤ The moderate, serious and extreme imbalance types have disappeared, indicating that the YUA has made certain achievements in ED and EE protection.

#### 5.3.2. The Comparison of Temporal and Spatial Variation between Provinces and Urban Agglomerations

**Figure 3:**
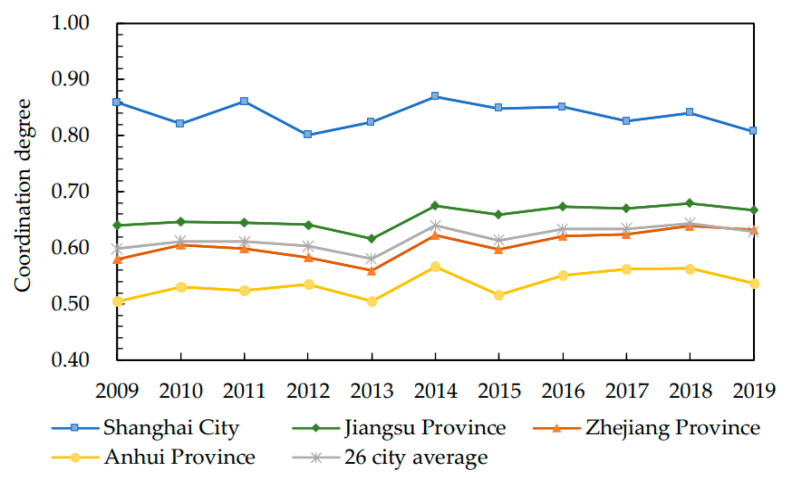
Changes in the Development Degree of Coordination between ED and EE in the YUA from 2009 to 2019.

**Figure 4:**
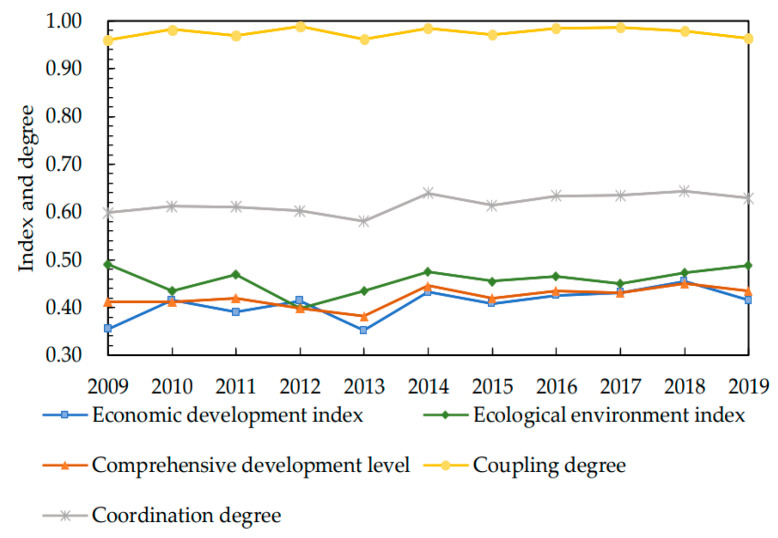
Data Changes of YUA from 2009 to 2019.

Based on the processing of data from cities, the weighted average values (hereinafter referred to as the average value) have been obtained and used as the data of provincial and urban agglomerations to study the development and change of the coordination degree between the ED and EE of the YUA and provinces (only the cities in the YUA included) (Figure 3 and Figure 4).

In Figure 3, Shanghai is way higher than Jiangsu, Zhejiang and Anhui in the development level of coordination at provincial (city) level, indicating that Shanghai is way ahead of other cities in the coordination level of ED and EE. As one of the first batch of coastal cities open to the world in China, Shanghai has rapidly become the economic center of China relying on its superior geographical position as the Yangtze River Delta and the supportive policies. Impacted by the economic crisis in 2008, Shanghai entered a period of medium-speed development before other cities in China. With the decline of economic growth, the scale effects reduced and its demand for resources was reduced. Specifically, its emission of industrial pollution was becoming controllable. After entering the post-industrialization stage, Shanghai was characterized by accelerated light industries, a plateau of heavy industries and stable and healthy development of high-tech industries. The growth rate of modern service industries is becoming much higher than that of traditional manufacturing industries, and the trend of industrial integration is becoming increasingly obvious. Meanwhile, through the releasing and implementation of a series of measures, such as the Negative List of Industrial Structure Adjustment and Energy Efficiency Guide, industrial pollution continued to decline, and the EE has been remarkably improved.

The coordination degree in Jiangsu Province and Zhejiang Province is close to the average level of the YUA. Jiangsu is slightly higher than the average level and Zhejiang is slightly lower, indicating that certain achievements in the ED and EE have been made in the two provinces. In 2019, in Jiangsu and Zhejiang, except Zhoushan, belonged to the top 100 cities in China, and the scale of Zhoushan’s ED was mainly limited by its natural conditions. At the time of achieving rapid economic growth, Jiangsu took the lead in formulating the policy of “priority to environmental protection” in China, which focuses on giving full play to the internal adjustment role of economic means while strengthening the necessary administrative means for external constraints. It stresses the internal adjustment of economic means and formulates and implements a series of environmental and economic policies. Jiangsu has also issued an action plan for the construction of ecological civilization and put forward six major actions, such as energy conservation and emission reduction, green growth, living environment with clear water and blue sky, green construction in Jiangsu, ecological protection and construction and the establishment of ecological demonstration. Based on the idea of sustainable development, Zhejiang focuses on promoting pollution reduction and intensive land use; it strengthens technological progress, management innovation, expansion in the space of resource and environment; and promotes the orderly construction of EE in the province from pollution reduction, environmental remediation, ecological construction and other fields.

Anhui Province has the lowest coordination level in the YUA, which accords with its development status in the agglomeration. Located far from the sea and affected by the siphonic effect on eastern developed cities, Anhui has a relatively low ED level. The industries in Anqing and other cities are mainly petrochemical and plastic industries, the development of which came at the expense of the environment, resulting in the low levels of EE. Meanwhile, as the main intake areas of industrial upgrading and transfer from the Yangtze River Delta, Anhui is faced with a difficult choice between ED and EE protection.

From the time dimension of the coordination degree of the three provinces, one city and urban agglomeration, the variation of their development generally showed an upward trend, but it fluctuated greatly in 2013 and 2014. In November 2012, the Chinese government formulated the strategic goal of promoting the five-sphere integrated plan in a new era, in which the construction of ecological civilization was raised to a higher level. In terms of economic development, we should establish an environment-friendly industry system, promote environment-friendly urbanization and form an environment-friendly production and consumption mode. In response to the call for ecological civilization, the YUA accelerated its industrial transformation and upgrading, and while vigorously developing high-end manufacturing industries and digital economy, the transfer of extensive industries was carried out in the region. Although the economic development had been negatively impacted by industrial transfer in a short time, the development of ecological economy will be conducive to the sustainable development of cities in the long-term perspective.

In Figure 4, after the five-sphere integrated plan was formulated in 2012, the ED index of the YUA declined briefly in 2013, but it quickly recovered to the original level and increased in 2014. The EE index has been increasing year by year since 2012, and declined after reaching its peak in 2014, but it showed an overall upward trend. This indicates that in the early stage of the releasing of these policies, city managers might have neglected the development in other fields in order to respond to the call of vigorously developing ecological civilization. Nevertheless, with the deepening of construction and the exploration of ecological civilization, the strategies of coordinated development were gradually improved, as well as the coordination degree and development level.

## 6. Conclusions

By establishing the evaluation system of ED and EE, the paper has used the grey relational model to determine the relational degree between the evaluation indicators of two systems and subsystems and analyzed the degree of the influence and correlation of these indicators. This paper uses the coupling coordination degree model to calculate the ED index, EE index, comprehensive development index and coupling coordination degree of the 26 cities in the YUA from 2009 to 2019. Our research found that since 2009, the coordination between the ED and EE of the YUA has experienced a stable growth period, which is closely related to the macro social background and relevant national policies.

There is still a gap between the economic and environmental development level of the 26 cities in the Yangtze River Delta urban agglomeration, and the imbalance in spatial distribution is serious. Under the background of implementing the regional integration and high-quality development strategy in the Yangtze River Delta, the coordinated development of cities should be designed from the following aspects: (1) Cities that belong to the ED lagging type should subdivide regional industries and strengthen urban cooperation. Meanwhile, they should take advantage of “The Belt and Road Initiative”, their location and openness and achieve high-quality and rapid economic growth. (2) Shanghai, as a city with lagging EE, should give full play to its leading role as a central city and drive the common development of its neighboring cities. Meanwhile, it should also strengthen the construction of the EE to coordinate with its ED. (3) For the balanced low-level cities, they should carry out differentiated urbanization positioning and choose the path that best suit themselves, so as to realize the rapid urban development. Meanwhile, the coordination between the economy and environment should also be prioritized to realize a win-win between growth rate and development quality. For the balanced and high-level cities, since there is still much room for their improvement, they should strive to achieve a higher level of balanced development on the premise of maintaining the current path of development.

The YUA is one of the six major urban agglomerations of the world, which takes lead in the high-quality coordinated development of the economy and environment in China. However, there is still a long way for its development level. Based on current economic condition, the YUA should use the advantages of complementary resources and spatial proximity, and the cities in the agglomeration should clarify their respective tasks and rely on and complement each other to establish a sustainable urban agglomeration system with global competitiveness.

The results of the determination and theoretical analysis will complement and improve the studies on cities and urban agglomerations, which will be beneficial to dealing with the contradiction between the economy and environment in the urbanization, promoting the integrated and high-quality development of urban agglomerations, and providing practical data analysis for the government and urban managers to adjust urban development strategy. Nevertheless, the paper has only explored the efficiency and quality of urban development from the aspects of the coupling coordination degree and relational degree between economic development and EE. More dimension shall be introduced to the analysis and the determination of the development process and the quality of the cities in the trend of diversified urban development.

## Figures and Tables

**Table 1:** Evaluation Index System for Coordinated Development of ED and EE in YUA.

System Level	Element Level	Indicator Level	Property	Weight
Economic development system	Scale of economic development	GDP	+	0.1736
		Total retail sales of consumer goods	+	0.1811
		Fixed asset investment	+	0.1075
	Economic development potential	GDP growth rate	+	0.0508
		proportion of tertiary industry in GDP	+	0.0689
		Growth rate of retail sales of consumer goods	+	0.0890
		Growth rate of fixed asset investment	+	0.0460
	Quality of economic development	GDP per capita	+	0.0834
		Annual balance of per capita deposit	+	0.1125
		Per capita fiscal revenue	+	0.0872
Eco-environmental system	Eco-environmental level	Per capita water supply	+	0.3373
		Per capita green area	+	0.0868
		Afforestation coverage rate of built-up areas	+	0.1563
	Eco-environmental system	Discharge of industrial wastewater	-	0.0616
		Industrial sulfur dioxide emissions	-	0.0569
		Industrial smoke (powder) dust emission	-	0.0898
	Eco-environmental protection	Centralized treatment rate of urban sewage	+	0.0888
		Rate of harmless disposal of urban household garbage	+	0.0509
		Comprehensive utilization rate of general industrial solid waste	+	0.0716

**Table 2:** Classification Criteria for the Degree of Correlation and Coupling Effect Level.

Number	(0,0.35]	(0.35,0.65]	(0.65,0.85]	(0.85,1]
Correlation level	Weak correlation	Medium correlation	Strong correlation	Extremely strong correlation

**Table 3:** Coupling Coordination Classification System and Discriminatory Criteria.

Interval	[0,0.1)	[0.1,0.2)	[0.2,0.3)	[0.3,0.4)	[0.4,0.5)	[0.5,0.6)	[0.6,0.7)	[0.7,0.8)	[0.8,0.9)	[0.9,1]
Coupling	Extreme Disordered	Serious Disordered	Moderate Disordered	Mild Disordered	On the verge Disordered	Barely Coordinated	Primary Coordinated	Intermediate Coordinated	Good Coordinated	Excellent Coordinated
Coordination										
Color										
Category	Disordered and declined				Transitional development		Coordinated development			
Interval	Unacceptable interval				Transitional interval		Acceptable interval			

**Table 4:** 2019 YUA ED System and EE System Relevance Ranking.

One	Indicator	Correlation	Two	Indicator	Relational Degree
1	Proportion of tertiary industry to GDP	0.6390	1	Discharge of industrial wastewater	0.6915
2	Per capita fiscal revenue	0.6342	2	Industrial smoke and dust emissions	0.6911
3	GDP growth rate	0.5775	3	Per capita water supply	0.6824
4	Per capita GDP	0.5762	4	Industrial sulfur dioxideemissions	0.6701
5	Annual balance of per capita deposit	0.5750	5	Per capita green area	0.5898
6	Growth rate of fixed asset investment	0.5750	6	Afforestation coverage rate of built-up areas	0.5545
7	Fixed asset investment	0.5741	7	Comprehensive utilization rate of general industrial solid waste	0.5277
8	GDP	0.5600	8	Centralized treatment rate of urban sewage	0.4670
9	Total retail sales of consumer goods	0.5549	9	Rate of harmless disposal of urban household garbage	0.3803
10	Growth rate of total retail sales of consumer goods	0.4820			

**Table 5:** Values and Ranks of ED and EE Subsystem Correlations in the YUA in 2019.

Indicator	Scale of Economic Development	Economic Development Potential	Quality of Economic Development	Average Correlation
EE level	0.6782 (Strong)	0.6217 (Moderate)	0.7156 (Strong)	0.6718
EE pressure	0.4721 (Moderate)	0.6652 (Strong)	0.5371 (Moderate)	0.5581
EE protection	0.6042 (Moderate)	0.4444 (Moderate)	0.5088 (Moderate)	0.5191
Average correlation	0.5848	0.5771	0.5872	

**Table 6:** Coordination between ED and EE in the YUA from 2009 to 2019.

City	2009	2010	2011	2012	2013	2014	2015	2016	2017	2018	2019
Shanghai	0.8586	0.8206	0.8608	0.8009	0.8240	0.8692	0.8489	0.8508	0.8254	0.8405	0.8075
Nanjing	0.7386	0.7335	0.7697	0.7578	0.7399	0.8012	0.7768	0.7798	0.7864	0.8100	0.8097
Wuxi	0.7148	0.7314	0.7314	0.6954	0.6626	0.6894	0.7031	0.7157	0.7313	0.7582	0.7154
Changzhou	0.6452	0.6488	0.6516	0.6330	0.5965	0.6924	0.6726	0.6976	0.7061	0.7018	0.6755
Suzhou	0.7736	0.7669	0.7599	0.7586	0.7780	0.7773	0.7612	0.7463	0.7650	0.7673	0.7628
Nantong	0.5986	0.6131	0.6096	0.6384	0.5737	0.6616	0.6527	0.6797	0.6445	0.6772	0.6540
Yancheng	0.4975	0.4819	0.4564	0.4615	0.4398	0.5485	0.5385	0.5549	0.5463	0.5375	0.5459
Yangzhou	0.5635	0.5846	0.5867	0.5878	0.5440	0.6119	0.6127	0.6229	0.6125	0.6355	0.6077
Zhenjiang	0.5873	0.6005	0.5994	0.6089	0.5729	0.6556	0.6113	0.6366	0.5725	0.5461	0.5730
Taizhou	0.5165	0.5312	0.4780	0.4834	0.4646	0.5292	0.4975	0.5462	0.5651	0.5495	0.5585
Hangzhou	0.7236	0.7292	0.7250	0.6997	0.6865	0.7570	0.7272	0.7307	0.7205	0.7420	0.7424
Ningbo	0.6102	0.6356	0.6438	0.6280	0.6140	0.6532	0.6418	0.6640	0.6648	0.6758	0.6841
Jiaxing	0.5549	0.5625	0.5829	0.5612	0.5396	0.6046	0.5745	0.5942	0.5837	0.5928	0.5848
Huzhou	0.5351	0.5558	0.5814	0.5703	0.5019	0.6056	0.5814	0.6162	0.6069	0.6523	0.6028
Shaoxing	0.5716	0.5933	0.5976	0.5706	0.5726	0.6509	0.6252	0.6409	0.6420	0.6601	0.6534
Jinhua	0.5153	0.5313	0.5345	0.5309	0.5000	0.5337	0.5277	0.5592	0.5523	0.5599	0.5643
Zhoushan	0.5486	0.5368	0.5463	0.5329	0.4906	0.545	0.5013	0.5478	0.5655	0.5738	0.6070
Taiizhou	0.5140	0.6400	0.5264	0.5212	0.5123	0.5712	0.5309	0.5719	0.6208	0.6162	0.5802
Hefei	0.5999	0.6185	0.6261	0.6020	0.5945	0.6633	0.6704	0.6238	0.6369	0.6621	0.6445
Wuhu	0.5305	0.5655	0.5728	0.5766	0.5204	0.5696	0.5201	0.5947	0.5805	0.5959	0.5687
Mananshan	0.5978	0.6040	0.6147	0.5950	0.5046	0.6062	0.5478	0.6093	0.6157	0.6389	0.5752
Tongling	0.5203	0.5990	0.5772	0.6237	0.5292	0.6386	0.5536	0.5824	0.5975	0.5413	0.5297
Anqing	0.4376	0.4650	0.4301	0.4560	0.3913	0.4872	0.3925	0.5002	0.5124	0.5035	0.4711
Chuzhou	0.3897	0.4328	0.4065	0.4850	0.4470	0.4737	0.4405	0.4675	0.4896	0.5258	0.5268
Chizhou	0.4763	0.4329	0.4099	0.4717	0.4378	0.5162	0.4483	0.5000	0.5193	0.5012	0.4611
Xuancheng	0.3766	0.4329	0.4210	0.3602	0.5490	0.5108	0.4439	0.4825	0.5020	0.4750	0.4556

## Data Availability

The data presented in this study are available on request from the corresponding author.
